# Effect of Asafoetida Extract on Growth and Quality of *Pleurotus ferulic*

**DOI:** 10.3390/ijms11010041

**Published:** 2009-12-29

**Authors:** Zuoshan Feng, Yujia Bai, Fanglin Lu, Wenshu Huang, Xinmin Li, Xiaosong Hu

**Affiliations:** 1 College of Food Science and Nutritional Engineering, China Agricultural University, Beijing 10083, China; E-Mails: fengzuoshan@126.com (Z.F.); lindalu101@yahoo.cn (F.L.); 2 College of Food Science Xinjiang Agricultural University, Ulumuqi, Xinjiang 830052, China; E-Mails: xjnd-hws@126.com (W.H.); saintbyj@126.com (Y.B.); 3 Gansu Agricultual Institute of Sciences, Lanzhou, Gansu 730010, China; E-Mail: lixinming4@126.com (X.L.)

**Keywords:** Pleurotus ferulic, asafoetida extract, amino acids, volatile components

## Abstract

Different concentrations of asafoetida extract were added to the medium of *Pleurotus ferulic* and the effects of the extract on growth of *P. ferulic* mycelium and fruiting bodies was observed. As the amount of asafoetida extract additive was increased, the growth of *Pleurotus* mycelium was faster, the time formation of buds was shorter and that yield of fruiting bodies was stimulated. However, overdosing of asafoetida extract hampered the growth of *Pleurotus ferulic*. The amino acid composition and volatile components in three kinds of pleurotus’ were contrasted, including wild pleurotus (WP), cultivated pleurotus with asafoetida extract (CPAE) and cultivated pleurotus without asafoetida extract (CP). CPAE with 2.3 g/100 g asafoetida extract addition had the highest content of total amino acids, as well as essential amino acids. WP had a higher content of total amino acids and essential amino acids than CP. In addition, CPAE with 2.3 g/100 g had the highest score of protein content of pleurotus fruiting bodies, while WP had a higher score than CP. In the score of essential amino acid components of pleurotus fruiting bodies, CP had the highest score, while CPAE was higher than WP. Asafoetida extract influenced the volatile components of *Pleurotus ferulic* greatly, making the volatile components of cultivated pleurotus more similar to those of wild pleurotus (WP).

## 1. Introduction

*Pleurotus ferulic* [1,2], whose Latin name is *Pleuratus eryngii* (Dc, ex, Fr)Quelet,belongs to Enmycophyta, Basidiomycetes, Agarieales, Pleurotaceae, Pleurotus. It mainly parasitizes or saprophytes the roots of samphire spp., like asafoetida (*Ferula sinkiangensis* K. M. Shen) [3]. The growth of cultivated pleurotus is affected not only by conditions like species, the environment and the nutrient contents of the medium, but also by some small organic molecules, like plant hormones, and secondary metabolites [4,5]. Wild *Pleurotus ferulic* as a parasitie or saprophyte in the roots and stems of asafoetida is highly undividedly. This kind of special relationship is thought to have developed due to long-time adaptation, evolution and heredity but the relationships establishing heredity have not been established. The components of asafoetida must influence the growth, nutrient, and flavor formation [6], although selected cultivated *Pleurotus ferulic* species can grow well without asafoetida, Researchers have investigated the influence of asafoetida content in the medium of *Pleurotus ferulic* on its growth and nutrients. The results showed that the *P. ferulic* with asafoetida powder had a relatively higher quality, that its mycelium grew faster and more exuberantly, and that its amino acids levels were apparently higher than without asafoetida powder [7]. Research on the influence of four kinds of Traditional Chinese Medicine samphire extracts including asafoetida, saposhnikovia, bupleurum, and nodakenin on *Braun pleurotus* K3 mycelium, showed that the appropriate concentrations of the four kinds of extract can all prompt the growth of *B. pleurotus* mycelium [8].

In this research asafoetida extract was added to the *Pleurotus* medium in order to examine the influence of the extract on the growth, amino acids and volatile components of *Pleurotus ferulic*, indicating its influence on the nutrients and flavor and providing a research foundation for the coordination of growth, quality enhancement, and flavor improvement of this product.

## 2. Results and Discussion

### 2.1. Effect of Asafoetida Extract on Pleurotus Mycelium Growth

According to Table 1, asafoetida extract can improve mycelium growth. Except for the 0.3 g/100 g asafoetida extract treatment, which showed little difference with CP, other treatments have all shown obvious effects. For example, in the 0.8 g/100 g treatment, mycelium grew to fill plastic bags (*i.e.,* grew to a length of 15.0 cm) after only 38 days, two days earlier than CP (40 days); in the 1.3 g/100 g and 1.8 g/100 g treatments, mycelium filled the plastic bags in both cases within 37 days, three days earlier that CP; in the 2.3 g/100 g and 2.8 g/100 g treatments, mycelium grew to fill plastic bags within 36 days, four days earlier that CP, while mycelium grew more thickly than in the case of CP; in the 3.3 g/100 g treatment, mycelium grew to fill the plastic bags earlier than CP by two days, however, the rate of growth and the quality and thickness of mycelium were not as good as seen for the 2.3 g/100 g and 2.8 g/100 g treatments. This means that within some specific concentrations, as the the amount of asafoetida extract additive increases, growth of *Pleurotus* mycelium was faster, but that an overdose of asafoetida extract can inhibit mycelium growth.

### 2.2. Effect of Asafoetida Extract on the Generation of Pleurotus Buds

Table 2 demonstrates that the generation of *Pleurotus* buds was obviously promoted by the increasing concentration of asafoetida extract from 0.3 g/100 g to 2.8 g/100 g. However, further research confirmed that asafoetida extract at a concentration of about 3.3 g/100 g resulted in the inhibition of generation of buds.

### 2.3. Effect of Asafoetida Extract on the Yield of Pleurotus Fruiting Bodies

Comparable fruiting body growth characteristics were observed in culture medium supplemented with asafoetida extract (Table 3). In culture medium, supplementation with asafoetida extract from 0.3 g/100 g to 2.3 g/100 g significantly promoted growth of *Pleurotus* fruiting bodies (Table 3), and a maximum yield of fruiting bodies was obtained at a concentration of about 2.3 g/100 g. The results showed that the asafoetida extract was closely associated or correlated (R = 0.895) with the growth of ies over most of the culture period.

### 2.4. Effect of Asafoetida Extract on the Amino Acids Components of Pleurotus Fruiting Body

We chose CPAE with 2.3 g/100 g extract for the experiment to assess *Pleurotus ferulic* amino acids, because CPAE with this extract concentration promoted mycelium and fruiting body growth, with high yields of fruiting bodies. This means CPAE with 2.3 g/100 g extract had higher bio-conversion efficiency and bio-conversion rate, increasing the accumulation of nutrients. It is necessary for us to analyze the amino acid proportion ratios of protein, an important nutrient, to make an assessment of protein levels.

As illustrated in Table 4, the amino acid assessment experimental results showed that total amino acid in *Pleurotus ferulic* fruiting bodies was the highest in CPAE with 2.3 g/100 g extract, following by WP, CP. However, amino acid composition after different treatments varies differently, as the did the content of seven amino acids Asp, Thr, Ser, Glu, Gly, Ala and Pro, which was higher in WP than in CP, followed by CPAE with 2.3/100 g extract; the content of Val in WP is higher than CPAE, followed by CP; the content of Lys in CPAE is the same as CP, but lower that WP.

The contents of eight other amino acids in CPAE is higher than CP and WP. The content of Arg in CPAE is higher than WP by 209.1% and higher than CP by 507.1%. The content of Met in CPAE is higher than WP by 298.9% and higher than CP by 66.9%. The content of Try in CPAE is higher than WP by 123.5% and higher than CP by 81%. The content of Ile in CPAE is higher than WP by 68.2% and higher than CP by 121.9%. The content of the seven essential amino acids assessed, except for Thr and Val, in CPAE with 2.3 g/100 g extract, is higher than CP and WP. The content of Thr in CPAE is lower than WP and CP. The content of Val in CPAE is lower than WP.

As illustrated in Table 5, the treatment with asafoetida extract showed a marked effect on percentage composition of essential amino acids. At the concentration of 2.3 g/100 g, asafoetida extract showed the highest content of total necessary amino acids (49.79%) followed by egg (49.7%), CP (43.20%), WP (40.74%) and the FAO/WHO recommendations (35%).

As illustrated in Table 6, scoring of protein content of *Pleurotus* fruiting body is the highest (70.2%) for asafoetida extract (2.3 g/100 g) followed by CP (57.7 %), and WP (43.3%). As illustrated in Table 7, scoring of necessary amino acids components of fruiting bodies is the highest (73.2%) in CP, followed by CPAE with 2.3 g/100 g extract (70.3%), and WP (55.8%).

Wild *Pleurotus ferulic* lives specially in the roots of asafoetida, decomposing it as its own nutrient and suggesting that there must be some chemicals there that affect its growth. In the experiment, *Pleurotus ferulic* was extracted with 95% ethanol and concentrated to give a complex mixture of chemical components. These chemical components play an important role in amino acid conversion, synthesis and accumulation. In the experiment, we added asafoetida extract to *Pleurotus ferulic* culture in appropriate proportions. When adding higher contents of asafoetida extract, the growth of *Pleurotus* mycelium and fruiting bodies was inhibited to some extent. This will definitely affect nutrient composition accumulation in fruiting bodies, and amino acid contents. We are most interested in some phenomena in the experiment. The content of some amino acids in CP is higher than CPAE and WP, while the content of some other amino acids is higher in CPAE than CP and WP, especially Arg and Met. In the experiment, the irregular change of *Pleurotus ferulic* amino acids must be in correlation with some chemicals in asafoetida extract. Now we can only speculate that these asafoetida extract chemicals stimulate or inhibit the physiology and/or metabolism of *Pleurotus,* or is used directly as a source of amino acids. Now the principle of the relationship is not clear.

### 2.5. Comparative Analysis of Volatile Components in Three Kinds of Pleurotus’

In order to identify putative active compounds present within the asafoetida extract, gas chromatography and MS were employed. The chemical composition of the extract is reported in Table 8. The extract from the CP group was characterized by a very high content of diethyl succinate (14.9%), 2-methylsuccinic acid 2-hydroxysuccinimide ester (27.29%), benzoic acid 2-octyl ester (10.17%), diisobutyl phthalate (11.99%) and hexadecanoic acid ethyl ester (11.91%). The extract from the treatment group (2.3 g/100 g) was characterised by a very high content of guaiol (34.01%), 2-methylsuccinic acid 2-hydroxysuccinimide ester (17.86%), and diisobutyl succinate (18.27%). The extract from the wild pleurotus group was characterised by a very high content of diethyl succinate (11.28%), guaiol (11.32%), diisobutyl succinate (22.78%), 3,3-dimethyl-2,4,5-3-thiooctane (12.79%), *n*-pentadecanoic acid ethyl ester (9.78%) and 9,12-octadecadienoic acid ethyl ester (9.25%). The major components thus displayed some differences, which were affected by the treatment with asafoetida extract. Smaller differences occurred between group treated by asafoetida extract and wild pleurotus ones.

## 3. Experimental Section

### 3.1. Materials

Asafoetida and *Pleurotus ferulic* were collected from Qinghe County, Xinjiang Province, China. The *Pleurotus ferulic* strain used in this study was isolated from Wild *Pleurotus ferulic* and has been maintained in the culture collection of the Laboratory of Microorganism Physiology, Xinjiang agricultural university, China. The asafoetida was identified as *Ferula sinkiangensis* K. M. Shen.

### 3.2. Chemicals

Cottonseed hull, wheat bran, corn flour, high pressure polypropylene culture bags, rings, lime powder (all bought on the market); amino acid standards (Sigma Corporation); citric acid, boric acid, hydrochloric acid, sodium hydroxide, lithium hydroxide (all AR; Beijing Reagent Factory).

### 3.3. Methods

#### 3.3.1. Preparation of Asafoetida Extract

The asafoetida was dried, pulverized and extracted (20.0 Kg) with 95% EtOH (60.0L) at 75 °C for 5 h under reflux, and the supernatant was removed. The combined supernatant was then concentrated to obtain asafoetida extract.

#### 3.3.2. Cultivation of *Pleurotus ferulic*

The culture material used for *Pleurotus ferulic* cultivation, was composed of 80% cotton seed hull, 15% wheat bran, 5% corn flour and 65% water. Plastic bags (9 cm × 20 cm × 0.04 cm) containing substrates (135 g) were pasteurized at 121 °C for 2 h (temperature ramp to 35 °C was about 3 h), allowed to cool for 1 h to ca. 35 °C, then inoculated with 0.5% (w/w) *Pleurotus ferulic* Strain. The inoculated substrates were incubated in an enclosed room at 18–25 °C for 2 days. To stimulate fruiting, fresh air was introduced to maintain <1,000 ppm CO_2_. Each treatment contains fifty plastic bags.

#### 3.3.3. Initial Formation Time of *Pleurotus* Buds

To observe the effects of asafoetida extract with different concentration on growth of *Pleurotus* mycelium and fruiting bodies, it was cultured on the above-mentioned culture material. Length of mycelium was examined once at an interval of 7 days for 35 days. After that, length of mycelium was examined twice at an interval of 7 days until it filled the whole plastic bags. Twenty days after that, time of formation and number of *Pleurotus* buds were recorded.

#### 3.3.4. Yield of *Pleurotus* of Each Treatment Batch

The fruiting bodies of *Pleurotus* were collected from every plastic bag after spore maturation. The fresh *Pleurotus* were air dried for several days in outdoor shade. Total weight of every treatment batch and average weight of every plastic bag were calculated.

#### 3.3.5. Analysis of Amino Acids Composition in Asafoetida *Pleurotus* Fruiting Bodies

The content moisture and total N were determined according to procedures described by the AOAC (1990). The content of amino acids was determined using an A-200 amino acid analyzer (INGOS, Prague, Czech Republic). The analytical procedure applied was in accordance with the recommendations of the manufacturer. The freeze-dried material was hydrolyzed in 6 M HCl for 24 h at 110 °C. After cooling, filtering and washing, the hydrolysate was evaporated in a vacuum evaporator at a temperature below 50 °C for sulfur-containing amino acids and below 60 °C for others amino acids, the dry residue being dissolved in a buffer of pH = 2.2. The prepared sample was analysed using the ninhydrin method. Buffers of pH 2.6, 3.0, 4.25, and 7.9 were used. The ninhydrin solution was buffered at pH 5.5. A column 370 mm in length was filled with Ostion ANB INGOS ionex (Czech Republic). The temperature of the column was 55–74 °C and that of the reactor was 120 °C. The determination of the sulfur-containing amino acids, methionine and cystine, was carried out by means of oxygenating hydrolysis, using a mixture of formic acid and hydrogen peroxide (9:1) at 110 °C for 24 h. After cooling, the sample was processed as with acid hydrolysis. Buffers of pH 2.6 and 3.0 were used; the temperature of the column was 60 °C and that of the reactor was 120 °C. The calculations were carried out according to the external standard.

The composition of amino acids was also expressed as g/16 g N to estimate the quality of the protein in fruiting body of the pleurotus by comparing it with the FAO/WHO (1970) pattern. On the basis of the amino acid composition, the CS index was calculated using the Amino Acid Content Of Foods and Biological Data on Proteins (1970) [9].The objective function was established [Equations (1) and (2)]:

(1) CS=(Ax)(Ee)100/(Ae)(Ex)

where Ax- necessary amino acids content (g) in tested protein; Ae- necessary amino acids total content (g) in tested protein; Ex - necessary amino acids content (g) in egg protein; Ee-necessary amino acids total content (g) in egg protein. The AAS index was estimated using the Bano method [10]:

(2) AAS=Ax(mg/g)100/Ae(mg/g)

where Ax-necessary amino acids content (g) in tested protein; Ae-necessary amino acids total content (g) in tested protein.

### 3.4. Gas Chromatography–Mass Spectroscopy (GC–MS) Analysis

Samples of volatile compounds were diluted in methyl *tert*-butylether (MTBE; 1:100) and analysed in an Agilent GC–MS apparatus equipped with an Rtx-5 SIL fused-silica capillary column (30 m × 0.25 mm internal diameter, 0.25 μm film thickness, Restek). Helium (0.8 mL/min) was used as a carrier gas. Samples were injected in the split mode at a ratio of 1:10–1:100. The injector was kept at 250 °C and the transfer line at 280 °C. The column was maintained at 50 °C for 2 min and then programmed to 260 °C at 5 °C/min and held for 10 min at 260 °C. The MS was operated in the EI mode at 70 eV, in the m/z range 42–350. The identification of the compounds was performed by comparing their retention indices and mass spectra with those found in the literature and supplemented by the Wiley & QuadLib 1607 GC–MS libraries. The relative proportions of the volatile constituents were expressed as percentages obtained by peak area normalization, all relative response factors being taken as one. The Kovat indices were determined from the retention times after co-injection with *n*-alkanes.

## 4. Conclusions

*Pleurotus ferulic* cultivation experiments indicated that as the the amount of asafoetida extract additive increased, growth of mycelium and the formation time of buds were faster, and that yield of *Pleurotus ferulic* fruiting bodies was enhanced. However, an overdose of asafoetida extract hampered the growth.

Results of amino acids analysis showed that CPAE with 2.3 g/100 g asafoetida extract addition had the highest content of total amino acids as well as essential amino acids. WP had a higher content of total amino acids and essential amino acids than CP. In addition, CPAE with 2.3 g/100 g had the highest score of protein content of *Pleurotus* fruiting bodies, while WP had a higher score than CP. In the score of necessary amino acids components of fruiting bodies, CP had the highest score, while CPAE was higher than WP.

The content of asafoetida had a great influence on the volatile components of *Pleurotus ferulic*, influencing the flavor, making the volatile components of CPAE more similar to that of WP, and providing an effective way for enhancing the quality and flavor of cultivated *Pleurotus ferulic*.

## Figures and Tables

**Table 1.** Effect of asafoetida extract on pleurotus mycelium growth.

**Treatment**	**7d**	**14d**	**21d**	**28**	**35d**	**36d**	**37d**	**38d**	**39d**	**40d**	**Average mycelium length (cm/d)**	**Growth condition**
0 g (CP)	0.3	3.6	6.8	9.4	11.8	12.3	12.8	13.6	14.4	15.0	0.375	+
0.3 g	0.3	3.6	6.9	10.0	12.7	13.2	13.7	14.1	14.6	15.0	0.375	+
0.8 g	0.4	3.7	6.9	10.0	12.9	13.5	14.4	15.0	15.0	15.0	0.395	++
1.3 g	0.3	3.6	6.8	10.5	13.9	14.5	15.0	15.0	15.0	15.0	0.405	+++
1.8 g	0.4	3.7	6.9	10.6	13.9	14.8	15.0	15.0	15.0	15.0	0.405	+++
2.3 g	0.5	3.8	7.0	10.7	14.0	15.0	15.0	15.0	15.0	15.0	0.417	++++
2.8 g	0.4	3.7	6.9	10.6	13.8	15.0	15.0	15.0	15.0	15.0	0.417	++++
3.3 g	0.4	3.7	6.9	9.6	12.9	13.8	14.5	15.0	15.0	15.0	0.395	+++

Date stands for the average level of each treatment; ++++, +++, ++, + means the growth of mycelium is diminishing.

**Table 2.** Effect of asafoetida extract on the generation and number of *Pleurotus* buds.

**Treatment**	**125d**	**128d**	**131d**	**134d**	**137d**	**140d**	**143d**	**146d**
0 g (CP)					14	36	45	50
0.3 g				12	22	38	50	50
0.8 g		1	10	30	46	50	50	50
1.3 g		2	12	24	40	50	50	50
1.8 g		1	7	25	28	42	46	50
2.3 g	1	6	11	24	45	50	50	50
2.8 g		5	19	31	40	50	50	50
3.3 g		4	4	14	26	47	50	50

**Table 3.** Effect of asafoetida extract on the yield of pleurotus fruiting bodies.

**Treatment**	**Average weight (g/bag)**	**Total weight (g)**	**Increased percent (%)**
0 g(CP)	8.2	410.0	-
0.3 g	10.0	500.0	21.95%
0.8 g	13.7	685.0	67.07%
1.3 g	14.3	715.0	74.39%
1.8 g	16.3	815.0	98.78%
2.3 g	19.6	980.0	139.02%
2.8 g	17.4	870.0	112.20%
3.3 g	17.4	870.0	112.20%

**Table 4.** Amino acids components of *Pleurotus* fruiting bodies.

**Amino acid**	**Content (amino acids g/100 g dry *Pleurotus*)**		
	**WP**	**CP**	**CPAE with 2.3 g/100 g extract**
Asp^**^	3.40 ± 0.03^A^	3.30 ± 0.03^A^	2.90 ± 0.07^B^
Thr^*^	1.10 ± 0.07^a^	0.90 ± 0.03^b^	0.85 ± 0.03^b^
Ser^**^	1.20 ± 0.03^A^	0.98 ± 0.04^A^	0.86 ± 0.01^B^
Glu^*^	3.40 ± 0.03^A^	3.20 ± 0.06^B^	3.10 ± 0.04^B^
Gly^**^	1.30 ± 0.04^A^	1.10 ± 0.03^B^	1.00 ± 0.04^B^
Ala^**^	2.20 ± 0.04^A^	1.50 ± 0.03^B^	1.30 ± 0.01^B^
Val^**^	1.30 ± 0.01^A^	0.75 ± 0.01^C^	0.96 ± 0.03^B^
Met^**^	0.06 ± 0.04^C^	0.14 ± 0.01^B^	0.23 ± 0.01^A^
Ile^**^	0.95 ± 0.03^B^	0.72 ± 0.03^C^	1.60 ± 0.04^A^
Leu^**^	1.70 ± 0.03^B^	1.40 ± 0.04^C^	2.40 ± 0.07^A^
Tyr^**^	1.70 ± 0.04^C^	2.10 ± 0.03^B^	3.80 ± 0.08^A^
Phe^*^	1.00 ± 0.06^A^	0.83 ± 0.03^B^	1.10 ± 0.03^A^
His^**^	0.66 ± 0.03^B^	0.53 ± 0.01^C^	0.88 ± 0.01^A^
Lys	1.40 ± 0.00^a^	1.10 ± 0.04^b^	1.10 ± 0.14^b^
Arg^**^	1.10 ± 0.04^B^	0.56 ± 0.01^C^	3.40 ± 0.04^A^
Pro^**^	0.72 ± 0.01^A^	0.53 ± 0.03^B^	0.33 ± 0.01^C^
Cys^**^	0.40 ± 0.03^C^	0.96 ± 0.08^B^	1.61 ± 0.04^A^
Trp	-	-	-
Total amino acids ^**^	23.589^B^	20.606^C^	27.423^A^

Unit: Amino acids g/100 g dry *Pleurotus*.

** Indicates that amino acid analysis of variance F test reached a significant difference at the 0.01 level.

* Indicates that amino acid analysis of variance F test reached a significant difference at the 0.05 level.

A, B, C indicates that there is no difference in the same characteristic when reaching a significant difference at 0.01.

a, b, c indicates that there is no difference in the same characteristic when reaching a significant difference at 0.05. Q test in least significant ranges is used in multiple comparisons.

**Table 5.** Essential amino acid components of pleurotus fruiting body.

**Amino acids**	**WP %**	**CP %**	**CPAE with 2.3 g/100 g extract %**	**Egg %**	**FAO/WHO %**
Thr	4.66	4.35	3.11	5.1	4
Val	5.51	3.66	3.52	7.3	5
Met + Cys	1.95	5.34	6.69	5.5	3.5
Ile	4.03	3.50	5.83	6.6	4
Leu	7.21	6.79	8.75	8.8	7
Phe + Tyr	11.45	14.23	17.87	10	6
Lys	5.93	5.34	4.01	6.4	5.5
Total content	40.74	43.20	49.79	49.7	35

**Table 6.** Scoring of protein content of *Pleurotus* fruiting bodies.

**Amino acids**	**WP**	**CP**	**CPAE with 3.3 g/100 g extract**
Thr	111.5	98.1	77.7
Val	92.1	57.7	70.2
Met + Cys	43.3	111.6	190.9
Ile	74.5	61.0	145.6
Leu	99.9	88.8	124.8
Phe + Tyr	139.6	163.7	297.3
Lys	113.1	96.0	72.8
Score of protein content	43.3	57.7	70.2

**Table 7.** Scoring of essential amino acid components of *Pleurotus* fruiting bodies.

**Amino acids**	**WP**	**CP**	**CPAE with 3.3 g/100 g extract**
Thr	116.6	108.7	77.9
Val	110.2	73.2	70.3
Met + Cys	55.8	152.4	191.2
Ile	100.8	87.5	145.9
Leu	103.0	97.1	125.0
Phe + Tyr	190.8	237.1	297.8
Lys	107.9	97.1	72.9
Score of essential amino acids components	55.8	73.2	70.3

**Table 8.** Comparative analysis of volatile components in three kinds of pleurotus by GC/MS.

**Number**	**Chemicals**	**CP**	**CPAE with 2.3 g/100 g extract%**	**WP%**
1	β-Pinene	0.38	0.82	0.07
2	Limonene	0.01	0.07	0.05
3	5,11-Diene-nordihydroguaiaretic acetate	—	0.04	0.03
4	β-Sitosterol	0.50	1.24	3.01
5	Diethyl succinate	14.9	3.99	11.28
6	Guaiol	—	34.01	11.32
7	2-Methylsuccinic acid-2 - hydroxysuccinimide ester	27.29	17.86	—
8	Diisobutyl succinate	0.03	18.27	22.78
9	Benzoic acid-2′-octyl ester	10.17	1.87	4.02
10	3,3-Dimethyl-2,4,5-3-thiooctane	0.59	5.58	12.79
11	*n*-Eicosane	2.48	0.25	0.54
14	Diisobutyl phthalate	11.99	1.00	2.11
12	*n*-Pentadecanoic acid ethyl ester	4.43	4.76	9.78
13	Hexadecanoic acid ethyl ester	11.91	1.76	3.55
14	9,12-Octadecadienoic acid ethyl ester	1.51	4.54	9.25
15	(Z)-9-Octadecenoic acid ethyl ester; ethyl cis-9-octadecenoate	0.30	0.54	1.16
16	cis-Octadeca-9,12-dienoic acid ethyl ester	0.42	0.13	0.26
17	Linoleic acid	2.62	0.15	0.36
18	(9*Z*,17*Z*)-Octadecadienal	0.01	0.86	1.80
19	9(*Z*),12(*Z*),15(*Z*)-Octadecatrienoic acid methyl ester	0.11	0.02	0.01

## References

[1] Huang NL Chinese Large Fungi Colorful Illustrations1st ed China Agriculture Press Beijing, China 1998 95 96

[2] Mao XL Economic Fungi of China1st ed Science Press Beijing, China 1998 30

[3] Shan RH Yu ML Flora of China Science Press Beijing, China 1992 55 85 117

[4] Li YL Chen BS Li RC Research on growth regulators on growth of asafoetida pleurotus Mycelium. Edible Fungi 2004 3 22 23

[5] Li GX Shao SG Li YJ Effect of Hormone on the growth and yield of pleurotus eryngii var.nebrodensis Chin. Edible Fungi 2004 23 37 38

[6] Tian HX Liu XG Jia XH Effect of Growth of pleurotus ferulae on adding powder of ferula sinkiangensis in culture medium Chin. Edible Fungi 2008 27 30 33

[7] Li ZH Fan YM Wu HQ Bing L Ferulic pleurotus cultivation comparative test compound content of ferulic Edible Fungi 2005 2 42 43

[8] Li M Jian ZY Li XW Influence of some samphire Chinese medicine extract on braun pleurotus mycelium growth J. Anhui Agri. Sci 2006 34 5499 5500

[9] FAO Amino Acid Content of Foods and Biological Data on Proteins FAO Rome, Italy 1970 24 5 6 5537267

[10] Bano Z Rajarathram S Pleurotus as a nutritious food Tropical Pleurotuss-Biological Nature and Cultivation Methods The Chinese University Press Beijing, China 1982 363 380

